# Supplementary material for: Neuropilin-2 and Its Transcript Variants Correlate with Clinical Outcome in Bladder Cancer
Source: Genes (Basel). 2021 Apr 9;12(4):550. doi: 10.3390/genes12040550 (PMC8070368; doi:10.3390/genes12040550)
Supplement: Supplementary file 1 [file genes-12-00550-s001.pdf]

Supplements

Neuropilin-2 and its transcripts correlate with clinical outcome in bladder cancer

Table S1: Primers and probes used in this study

**Gene**	**Forward Primer Sequence (5 → 3`)**	**Reverse Primer Sequence (5` → 3`)**	**Probe Sequence (5`label → 3`label)**
CALM2	GAGCGAGCTGAGTGGTTGTG	AGTCAGTTGGTCAGCCATGCT	VIC-TCGCGTCTCGGAAACCG GTAGC-BHQ1
NRP1	CCCCAAACCACTGATAACTCG	GTCCAGACACCATACCCAACAT	FAM-CGAATCAAGCCTGCAACT TGGGAAACTGG-BHQ1
NRP2	GGGGGACTGCAAGTACGATT	AGAGGATCCCCGTCGATGAA	FAM-CTGGACATCTGGGATGGCA TTCCACATG-BHQ1
NRP2a	GGAGAACTGCATGGAACCCA	AATTGCTCCAGTCCACCTCG	FAM-CATCATCAATTTCATCTTC ATATCCTTCTCTCTCATG-BHQ1
NRP2b	ATGTCCCACTGGAGAACTGC	ATACCAGTAGGCTGGGATGGG	FAM-GCATGGGCACCGTGTCC ACTGTGG-BHQ1
PDGFC	TCCTGGTTAAACGCTGTGGT	GACACCGGTCTTTGGTCTCA	FAM-CTGTGCCTGTTGTCTCC ACAATTGCAATGAATG-BHQ1
PDGFD	TGGAAGATTTCCAACCCGCA	GCAATCAGAGTGGGATCCGT	FAM-CCAACTGGGAATCTGTCA CAAGCTCTATTTC-BHQ1

Table S2: NRP2 transcript variant expression in TCGA cohort

**Variant** (n)	Median (IQR)
**NRP2A** (360)	**8.83 (7.55-9.87)**
NRP2 A22 (120)	5.10 (0-7.38)
NRP2 A17 (285)	8.39 (6.64-9.47)
NRP2 A0 (211)	4.46 (0-6.31)
**NRP2B** (357)	**5.61 (3.99-6.94)**
NRP2 B5 (70)	0 (0-4.18)
NRP2 B0 (335)	5.34 (3.67-6.77)
**NRP2 S9** (243)	1.84 (0-3.06)

Abbreviations: IQR, interquartile range

Table S3: Ratio of NRP2A/NRP2B transcript variants in TCGA and the Mannheim cohort

NRP2A/NRP2B	>1	≤1
TCGA cohort (n, %)	360 (100)	0 (0)
Mannheim cohort (n, %)	95 (93.1)	7 (6.9)

Table S4: Correlation of NRPs and indicated ligands in TGCA cohort

Marker		NRP2	NRP2A	NRP2B	PDGFC	PDGFD
**NRP1**	ρ	0.6391	0.6451	0.6417	0.5376	0.1461
	P value	**<0.0001**	**<0.0001**	**<0.0001**	**<0.0001**	**0.0079**
**NRP2**	ρ		0.9977	0.9346	0.7396	0.1243
	P value		**<0.0001**	**<0.0001**	**<0.0001**	**0.0241**
**NRP2A**	ρ			0.9362	0.7394	0.1239
	P value			**<0.0001**	**<0.0001**	**0.0246**
**NRP2B**	ρ				0.6773	0.0549
	P value				**<0.0001**	0.3209
**PDGFC**	ρ					0.2507
	P value					**<0.0001**
**PDGFD**	ρ					
	P value					

Abbreviations: ρ, Spearman correlation

Table S5: Correlation of NRPs and indicated ligands in the Mannheim cohort

Marker		NRP2	NRP2A	NRP2B	PDGFC	PDGFD
**NRP1**	ρ	0.7272	0.6985	0.5816	0.7624	0.6764
	P value	**<0.0001**	**<0.0001**	**<0.0001**	**<0.0001**	**<0.0001**
**NRP2**	ρ		0.8413	0.6684	0.6869	0.6730
	P value		**<0.0001**	**<0.0001**	**<0.0001**	**<0.0001**
**NRP2A**	ρ			0.5360	0.6659	0.6268
	P value			**<0.0001**	**<0.0001**	**<0.0001**
**NRP2B**	ρ				0.5832	0.5525
	P value				**<0.0001**	**<0.001**
**PDGFC**	ρ					0.5226
	P value					**<0.0001**

Abbreviations: ρ, Spearman correlation

Table S6: Univariable and multivariable Cox regression analyses to test the effect of different parameters on disease-free survival in the TCGA cohort

	**Univariable analysis**			**Multivariable analysis**		
Variable	HR	95% CI	P value	HR	95% CI	P value
**Age** (high/low)	1.24	0.86-1.78	0.2432			
**Gender** (male/female)	0.91	0.62-1.37	0.6507			
**T stage** (T3/4 / T2)	2.59	1.69-4.11	**<0.0001**	2.05	1.13-3.98	**0.0170**
**N stage** (pos/neg)	2.42	1.67-3.53	**<0.0001**	1.48	0.89-2.78	0.1292
**LVI** (yes/no)	2.31	1.50-3.63	**0.0001**	1.53	0.89-2.64	0.1265
**NRP1** (high/low)	1.61	1.04-2.41	**0.0327**	1.35	0.77-2.28	0.2876
**NRP2** (high/low)	2.29	1.59-3.27	**<0.0001**	2.41	0.38-9.71	0.3101
**NRP2A** (high/low)	2.23	1.55-3.19	**<0.0001**	0.67	0.17-4.15	0.6163
**NRP2B** (high/low)	2.23	1.54-3.20	**<0.0001**	1.32	0.71-2.43	0.3788
**PDGFC** (high/low)	1.66	1.15-2.38	**0.0072**	0.90	0.49-1.64	0.7342
**PDGFD** (high/low)	1.48	1.02-2.13	**0.0389**	1.67	1.02-2.63	**0.0332**

Abbreviations: HR, hazard ratio; CI, confidence interval; LVI, lymphovascular invasion

Table S7: Associations of ABSOLUTE and ESTIMATE scores with T stage

	**ABSOLUTE**	**ESTIMATE**
Variable (n)	Median (IQR)	Median (IQR)
**T stage**		
T2 (107)	0.71 (0.5-0.86)	-1342.46 (-2329.44-413.94)
T3/4 (215)	0.58 (0.39-0.76)	-400.3 (-1863.14-980.47)
p value	**0.0010**	**0.0013**
